# Genotypic and Functional Impact of HIV-1 Adaptation to Its Host Population during the North American Epidemic

**DOI:** 10.1371/journal.pgen.1004295

**Published:** 2014-04-24

**Authors:** Laura A. Cotton, Xiaomei T. Kuang, Anh Q. Le, Jonathan M. Carlson, Benjamin Chan, Denis R. Chopera, Chanson J. Brumme, Tristan J. Markle, Eric Martin, Aniqa Shahid, Gursev Anmole, Philip Mwimanzi, Pauline Nassab, Kali A. Penney, Manal A. Rahman, M.-J. Milloy, Martin T. Schechter, Martin Markowitz, Mary Carrington, Bruce D. Walker, Theresa Wagner, Susan Buchbinder, Jonathan Fuchs, Beryl Koblin, Kenneth H. Mayer, P. Richard Harrigan, Mark A. Brockman, Art F. Y. Poon, Zabrina L. Brumme

**Affiliations:** 1 Faculty of Health Sciences, Simon Fraser University, Burnaby, British Columbia, Canada; 2 Department of Molecular Biology and Biochemistry, Faculty of Science, Simon Fraser University, Burnaby, British Columbia, Canada; 3 Microsoft Research, Los Angeles, California, United States of America; 4 KwaZulu-Natal Research Institute for Tuberculosis and HIV, Nelson R. Mandela School of Medicine, University of KwaZulu-Natal, Durban, KwaZulu-Natal, South Africa; 5 British Columbia Centre for Excellence in HIV/AIDS, Vancouver, British Columbia, Canada; 6 Faculty of Medicine, University of British Columbia, Vancouver, British Columbia, Canada; 7 Aaron Diamond AIDS Research Center, The Rockefeller University, New York, New York, United States of America; 8 Cancer and Inflammation Program, Laboratory of Experimental Immunology, Leidos Biomedical Research Inc., Frederick National Laboratory for Cancer Research, Frederick, Maryland, United States of America; 9 Ragon Institute of MGH, MIT and Harvard University, Cambridge, Massachusetts, United States of America; 10 San Francisco Department of Public Health, San Francisco, California, United States of America; 11 New York Blood Center, New York, New York, United States of America; 12 Fenway Community Health, Boston, Massachusetts, United States of America; 13 Harvard Medical School, Boston, Massachusetts, United States of America; Stanford University, United States of America

## Abstract

HLA-restricted immune escape mutations that persist following HIV transmission could gradually spread through the viral population, thereby compromising host antiviral immunity as the epidemic progresses. To assess the extent and phenotypic impact of this phenomenon in an immunogenetically diverse population, we genotypically and functionally compared linked HLA and HIV (Gag/Nef) sequences from 358 historic (1979–1989) and 382 modern (2000–2011) specimens from four key cities in the North American epidemic (New York, Boston, San Francisco, Vancouver). Inferred HIV phylogenies were star-like, with approximately two-fold greater mean pairwise distances in modern versus historic sequences. The reconstructed epidemic ancestral (founder) HIV sequence was essentially identical to the North American subtype B consensus. Consistent with gradual diversification of a “consensus-like” founder virus, the median “background” frequencies of individual HLA-associated polymorphisms in HIV (in individuals *lacking* the restricting HLA[s]) were ∼2-fold higher in modern versus historic HIV sequences, though these remained notably low overall (*e.g.* in Gag, medians were 3.7% in the 2000s versus 2.0% in the 1980s). HIV polymorphisms exhibiting the greatest relative spread were those restricted by protective HLAs. Despite these increases, when HIV sequences were analyzed as a whole, their total average burden of polymorphisms that were “pre-adapted” to the average host HLA profile was only ∼2% greater in modern versus historic eras. Furthermore, HLA-associated polymorphisms identified in historic HIV sequences were consistent with those detectable today, with none identified that could explain the few HIV codons where the inferred epidemic ancestor differed from the modern consensus. Results are therefore consistent with slow HIV adaptation to HLA, but at a rate unlikely to yield imminent negative implications for cellular immunity, at least in North America. Intriguingly, temporal changes in protein activity of patient-derived Nef (though not Gag) sequences were observed, suggesting functional implications of population-level HIV evolution on certain viral proteins.

## Author Summary

Upon HIV transmission, many – though not all – immune escape mutations selected in the previous host will revert to the consensus residue. The persistence of certain escape mutations following transmission has led to concerns that these could gradually accumulate in circulating HIV sequences over time, thereby undermining host antiviral immune potential as the epidemic progresses. As certain immune-driven mutations reduce viral fitness, their spread through the population could also have consequences for the average replication capacity and/or protein function of circulating HIV sequences. Here, we characterized HIV sequences, linked to host immunogenetic information, from patients enrolled in historic (1979–1989) and modern (2000–2011) HIV cohorts from four key cities in the North American epidemic. We reconstructed the epidemic's ancestral (founder) HIV sequence and assessed the subsequent extent to which known HIV immune escape mutations have spread in the population. Our data support the gradual spread of many - though not all - immune escape mutations in HIV sequences over time, but to an extent that is unlikely to have major immediate immunologic consequences for the North American epidemic. Notably, *in vitro* assessments of ancestral and patient-derived HIV sequences suggested functional implications of ongoing HIV evolution for certain viral proteins.

## Introduction

Escape from Human Leukocyte Antigen (HLA) class I-restricted CD8+ T-lymphocytes (CTL) in Human Immunodeficiency Virus Type 1 (HIV) occurs along mutational pathways that are broadly reproducible based on the HLA alleles expressed by the host [1]–[4]. The opposite phenomenon (that is, reversion of escape mutations to consensus upon HIV transmission to an individual lacking the restricting HLA) is somewhat more variable. While some escape mutations revert relatively rapidly following transmission [5]–[7], others do so more slowly [8], [9]. Yet others (perhaps because they harbor no fitness costs, or such costs are rescued by the presence of compensatory mutations) revert rarely or not at all [10]–[13]. If escape mutations reverted rapidly and consistently, their prevalence in HLA-mismatched persons would remain stably low (or negligible) over time [9]. However, escape mutations persisting upon transmission could gradually spread throughout the population [10], [12], [14]–[16]. Analogous to the negative impact of transmitted drug resistance mutations on treatment efficacy [17], acquisition of “immune escaped” HIV by persons expressing the relevant HLA allele could undermine the ability of their CTL to control infection. As such, the spread of HIV strains harboring escape mutations throughout the population could gradually undermine host antiviral immune potential, and potentially diminish the protective effects of certain HLA alleles, as the epidemic progresses [11], [12], [18].

The extent to which immune escape mutations are accumulating in HIV sequences over time remains incompletely elucidated – a knowledge gap attributable in part to the scarcity of historic data. Nevertheless, some supportive data exist. It has been suggested that CTL epitopes in European HIV sequences are being “lost” over time through mutational escape, in particular via selection by HLA-B alleles, though this study was limited by the modest number of sequences analyzed [19]. Higher HIV polymorphism frequencies have been reported in modern compared to historic South American HIV subtype B and F sequences, though this study was limited by the lack of host HLA characterization [20]. The high (∼75%) frequency of the B*51-associated HIV Reverse Transcriptase (RT) I135X mutation in Japan, a population where B*51 prevalence approaches 20%, is also consistent with escape mutation accumulation [12], though the possibility that the Japanese epidemic was founded by an HIV sequence containing RT-I135X cannot be ruled out. That certain (though not all) escape mutations are capable of spreading in HIV-infected populations has also been demonstrated via mathematical modeling [9]. However, conclusive assessment of the extent to which escape mutants are accumulating in circulation ideally requires large datasets of linked HLA/HIV genotypes from historic and modern eras, combined with ancestral (founder) sequence reconstruction of the studied epidemics.

The potential pathogenic implications of population-level HIV evolution are also of interest. It has been hypothesized that conflicting selection pressures imposed on HIV by HLA-diverse host populations could lead to (relative) viral attenuation over time, while consistent pressures imposed by populations with limited HLA diversity could increase HIV virulence [21]. However, the complex tradeoffs between immune evasion benefits versus fitness costs of escape, and the context-specific nature of these factors with respect to the host genetic milieu, render this a challenging question to address. A recent meta-analysis of HIV clinical prognostic markers (plasma viral load and CD4+ T-cell counts) in cohorts from North America, Europe and Australia suggested that HIV could be increasing in virulence [22], but other reports have been highly conflicting [23]–[30]. Alternatively, pathogenic implications may be investigated, albeit incompletely and indirectly, via assessment of HIV protein function and/or replication capacity of patient-derived viral sequences – though historic data remain scarce. Reductions in replication capacity of recombinant HIV expressing gag-protease sequences from Japanese patients, a population with relatively constrained HLA diversity [12], [31], have been reported since the 1990s [32], while two earlier studies examining replicative fitness of recombinant viruses expressing HIV RT sequences from historic and modern European isolates yielded opposing results [23], [33].

The goals of the present study are to assess the extent to which HLA-associated polymorphisms are accumulating in HIV sequences over time in a large epidemic region comprising an immunogenetically diverse population (North America), and to investigate whether any genotypic changes have been accompanied by functional implications for the virus. To do this, we genotypically and functionally assessed HIV sequences, linked to host HLA information, from 358 historic (1979–1989) and 382 modern (2000–2011) specimens from four key cities in the epidemic (New York [34], [35], Boston [36], [37], San Francisco [34], [38], [39] and Vancouver, Canada [40]–[42]). We performed ancestral phylogenetic reconstructions to infer North America's most recent common ancestor (MRCA) HIV sequence, and we defined HLA-associated polymorphisms based on independent published sources [43]. We focused on Gag and Nef, as these are immunogenic HIV proteins whose sequence variability is substantially influenced by HLA [43] and whose function is susceptible to immune-mediated attenuation [44]–[46].

Overall, we observed an HIV epidemic that is steadily diversifying (in part due to HLA pressures), where background frequencies of HLA-associated polymorphisms have, on average, increased by a modest extent over the study period. Notably, HIV polymorphisms selected by protective HLA alleles appear to have increased to a greater relative (though not absolute) degree than those restricted by non-protective alleles. Despite these increases, average escape mutation background frequencies remain, in absolute terms, low. As such, we contend that HIV adaptation to host HLA is unlikely to yield imminent negative implications for cellular antiviral immunity, at least in North America. Intriguingly, changes in Nef (though not Gag) activity were observed over the epidemic's course, suggesting functional impacts of ongoing HIV evolution on certain viral proteins.

## Results

### HLA and HIV diversity in historic and modern cohorts

A total of 358 historic HIV sequences spanning 1979–1989, from observational cohorts of men who have sex with men (MSM) established in four key cities in the North American epidemic (New York [34], [35], Boston [36], [37], San Francisco [34], [38], [39] and Vancouver [40]–[42]), were studied alongside 382 modern North American HIV sequences spanning 2000–2011 from untreated persons belonging to various risk groups. High-resolution HLA class I sequence-based typing, aided where necessary by imputation using a published [47] and extensively validated [43] machine-learning algorithm, was successful for 330 (of 358; 92.2%) historic and 381 (of 382; 99.7%) modern specimens. The lower success rate for historic samples reflects the use of serum or plasma as a genomic DNA source [48]. A limitation of serum-based typing is the potential overrepresentation of homozygous types due to amplification of only one allele of the pair [48]; indeed, this bias was noted (*e.g.*: HLA-B homozygosity was 9% in the historic compared to 5% in the modern cohort, p = 0.03). Nevertheless, historic and modern cohorts exhibited comparable HLA allele frequencies (Pearson's R = 0.97, p<0.0001, and **Figure S1**), indicating that our analyses of the spread of HLA-associated HIV polymorphisms are unlikely to be majorly confounded by intercohort differences in the frequencies of their restricting HLA alleles.

Plasma HIV RNA amplification and bulk sequencing of Gag and/or Nef was successful for the above-mentioned 358 historic specimens (of an original total of 497 specimens tested, 72.0% genotyping success rate), yielding 299 Gag and 335 Nef sequences for study. Success rates of historic Gag and/or Nef genotyping, by site, were: New York 73 (of 94; 77.6%), San Francisco 32 (of 75; 42.7%), Boston 242 (of 282; 85.8%) and Vancouver 11 (of 46, 23.9%). Infection stage was unknown for most historic specimens, though these included 67 individuals with known or suspected early infection, all from New York. Gag and/or Nef sequencing was successful for 382 modern specimens in total: 358 (93.7%) for Gag and 337 (88.2%) for Nef, all from individuals with chronic infection. All HIV sequences were subtype B.

**Figure 1:**
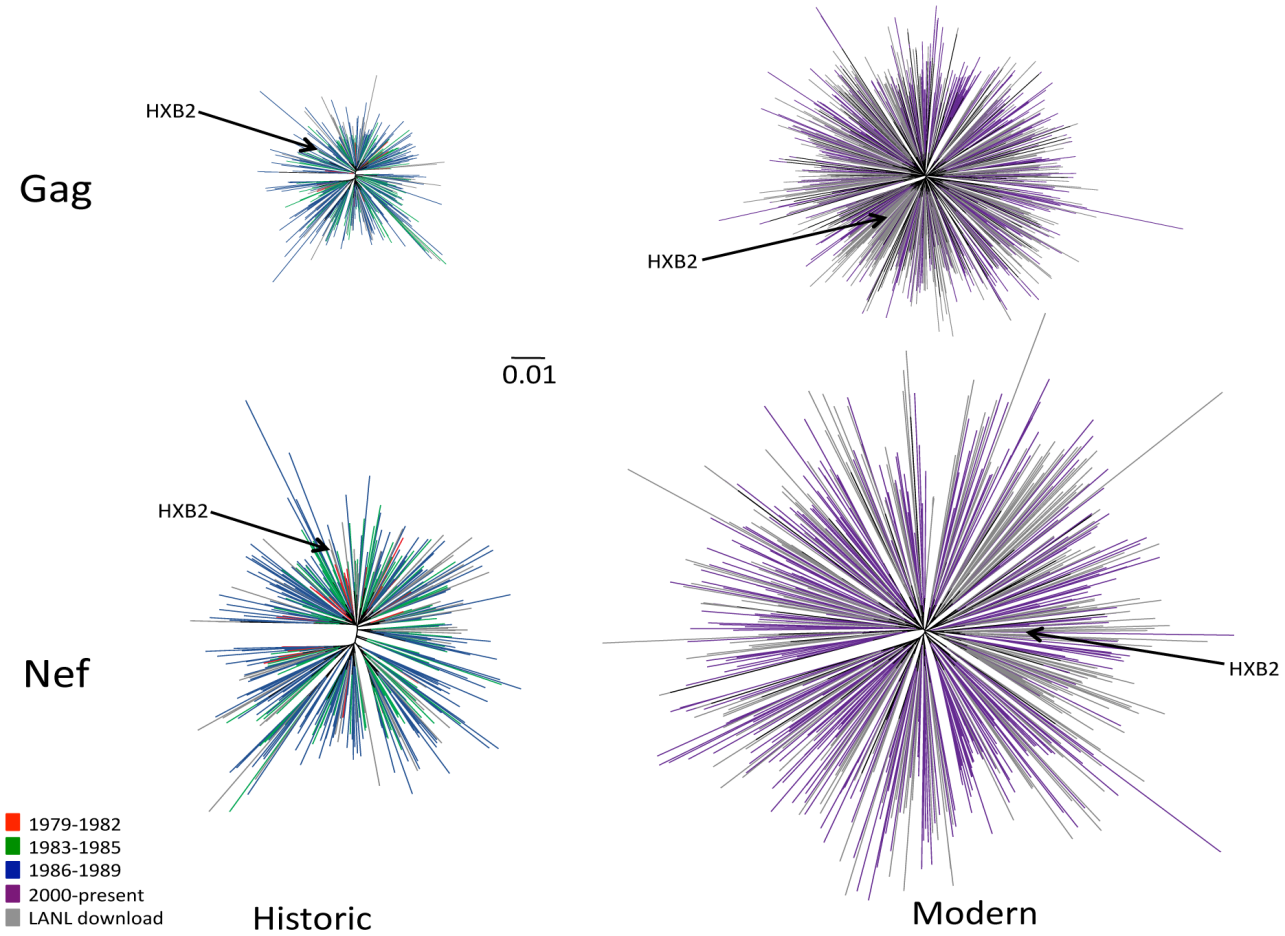
Diversity of North American Gag and Nef sequences from historic (1979–1989) and modern (2000+) eras. Unrooted Maximum likelihood phylogenetic trees, drawn on the same distance scale, are shown for historic Gag (upper left), historic Nef (lower left), modern Gag (upper right) and modern Nef (lower right). Phylogenies are star-like, with Nef exhibiting greater diversity than Gag, and modern trees exhibiting greater diversity than historic ones. Cohort sequences are colored by sampling era: red (1979–1982), green (1983–1985), blue (1986–1989) and purple (2000+); North American sequences retrieved from the Los Alamos (LANL) database are in grey. Included in each tree is the HIV subtype B reference strain HXB2, shown in black and indicated with an arrow.

Estimated maximum-likelihood HIV Gag and Nef phylogenies exhibited star-like shapes typical of HIV sequences sampled from a population [49] (**Figure 1**). Despite being a convenience sample, historic sequences exhibited no gross segregation by early (1979–1982; N = 28), mid (1983–1985; N = 122) and later (1986–1989; N = 208) eras. Moreover, unique historic North American sequences in the Los Alamos National Laboratory (LANL) HIV database (totaling 27 Gag and 56 Nef sequences spanning 1982–1989) were interspersed throughout the phylogenies, as were sampled modern LANL sequences spanning 2000-present (**Figure 1**). Despite some clustering by city and the predominance of historic sequences in two lineages of a combined phylogeny (**Figure S2**), the historic and modern cohort consensus HIV sequences were consistent with one another as well as the LANL North American and global (worldwide) subtype B consensus sequences (**Figure S3**), with all differences occurring at highly variable residues. Results thus support our HIV sequences as not grossly unrepresentative of the North American epidemic.

HIV sequence diversity within the modern cohort was substantially greater than that of the historic cohort (**Figure 1**). Grouped by era, the mean (±standard deviation [SD]) patristic (pairwise) genetic distances in Gag were 0.020±0.004 (1979–1982), 0.027±0.009 (1983–1985), 0.034±0.009 (1986–1989), and 0.074±0.012 (2000+) substitutions per nucleotide site, while those for Nef were 0.043±0.010, (1979–1982), 0.057±0.014 (1983–1985), 0.072±0.015 (1986–1989), and 0.12±0.025 (2000+) substitutions per nucleotide site. Modern HIV cohort sequences (all sampled during chronic infection) exhibited comparable mean pairwise distances to modern acute-phase subtype B sequences not included in the previous analysis (not shown), suggesting that infection stage was not a major confounder of our diversity estimates by era. Taken together, results support a diversifying North American epidemic [50] where average intra-subtype Gag and Nef genetic distances have increased approximately two-fold since the 1980s.

### North American Gag and Nef MRCA sequences are essentially identical to consensus

**Figure 2:**
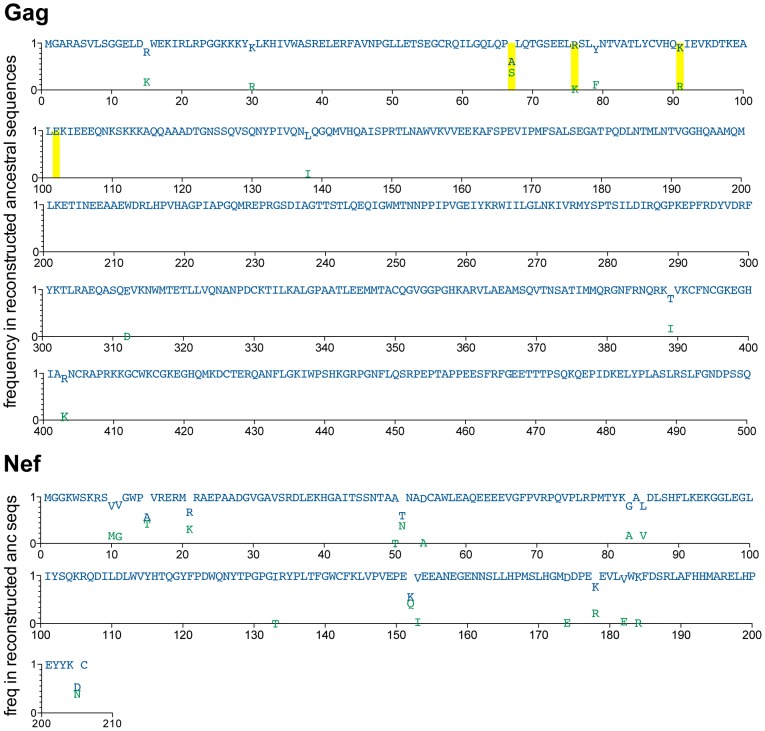
Reconstructed ancestral sequences at the root of the inferred Gag and Nef phylogenies, representing the estimated most recent common ancestor (MRCA) of the North American epidemic. A minimum of ≥50,000 reconstructions of the ancestral sequence at the root of the Gag and Nef phylogenies were performed, and the inferred MRCA was computed as the “grand consensus” of these replicate reconstructions. For each codon, reconstruction confidence (computed as the frequency of each amino acid observed across all reconstructions) is indicated on the y-axis on a scale from 0 (0%) to 1 (100%). Blue letters represent the highest-confidence residue at each position; green letters represent lower-confidence residues. All amino acids observed at >0.01 (>1%) reconstruction frequency are shown. Yellow boxes highlight positions where the highest-confidence (blue) inferred ancestral residue differs from the North American consensus B sequence (displayed in **Figure S3**).

Before claiming that any highly prevalent HIV polymorphism has arisen as a result of its spread through the population over time, it is important to rule out its presence at the epidemic's genesis (*i.e.* founder effect [51]). We therefore estimated the founder virus sequence of the North American epidemic by reconstructing the most recent common ancestor (MRCA) sequence at the root of the Gag and Nef phylogenies. To this end, we performed ≥50,000 MRCA reconstructions per HIV protein on random subsets of the historic sequence data using BEAST (see methods and [52]), and computed a “grand consensus” MRCA reconstruction per protein (**Figure 2**). Overall, reconstruction confidence exceeded 80% for all but one codon in Gag (residue 67) and for all but 6 codons in Nef (residues 15, 21, 51, 152, 178 and 205), all of which are highly polymorphic sites (<70% amino acid conservation) (**Figure 2**). The consensus of Gag sequence reconstructions at the MRCA differed from the LANL North American HIV subtype B consensus at only four residues (A67S, R76K, K91R and E102D), while the consensus of Nef MRCA reconstructions was identical to it (**Figures 2** **and S3**). Note the four ancestor/consensus differences in Gag merit cautious interpretation, as codon 67 was reconstructed with <80% confidence and the remainder are sites with <60% conservation at the amino acid level. MRCA reconstructions undertaken using random subsamples of both historic and modern Gag and Nef sequences were consistent with those computed from historic sequences only (not shown). Finally, the grand mean MRCA date estimate from phylogenetic reconstructions inferred from random subsamples of both historic and modern sequences was 1965 (range 1962–1967). The consistency of this date with published estimates of a 1960s U.S. epidemic origin [53]–[55] provides additional support for our data as representative of the North American epidemic.

### HIV diversification is attributable, at least in part, to HLA selection pressures

A diversifying epidemic will, by definition, feature increasing viral polymorphism frequencies. Thus, to give relevance to our objective of measuring the spread of HLA-driven polymorphisms in HIV sequences over time, it is important to first demonstrate that HIV diversification is driven, at least in part, by HLA pressures. If so, we reasoned that HIV codons known to be under selection by HLA would, on average, have diversified to a greater extent than those not under selection by HLA.

To investigate this, we first needed to independently define a list of HIV sites that are known to be under selection by specific HLA alleles. We defined these based on an independent published study of >1800 treatment-naïve individuals with chronic HIV subtype B infection from cohorts in Canada, the USA and Australia [43], that had no overlap with the historic or modern cohorts studied here. In that study, HLA-associated polymorphisms in HIV were identified using phylogenetically-corrected association testing approaches (see methods and [43]). For the present analysis of HLA selection and HIV diversification, an inclusive definition of “HIV sites under selection by HLA” was warranted; therefore, we defined this as all Gag and Nef codons associated with at least one HLA allele that met a false-discovery rate threshold of <20% (q-value <0.2) in the independent study (see methods and [43]). This totaled 95 (of 500) codons in Gag and 99 (of 206) codons in Nef.

**Figure 3:**
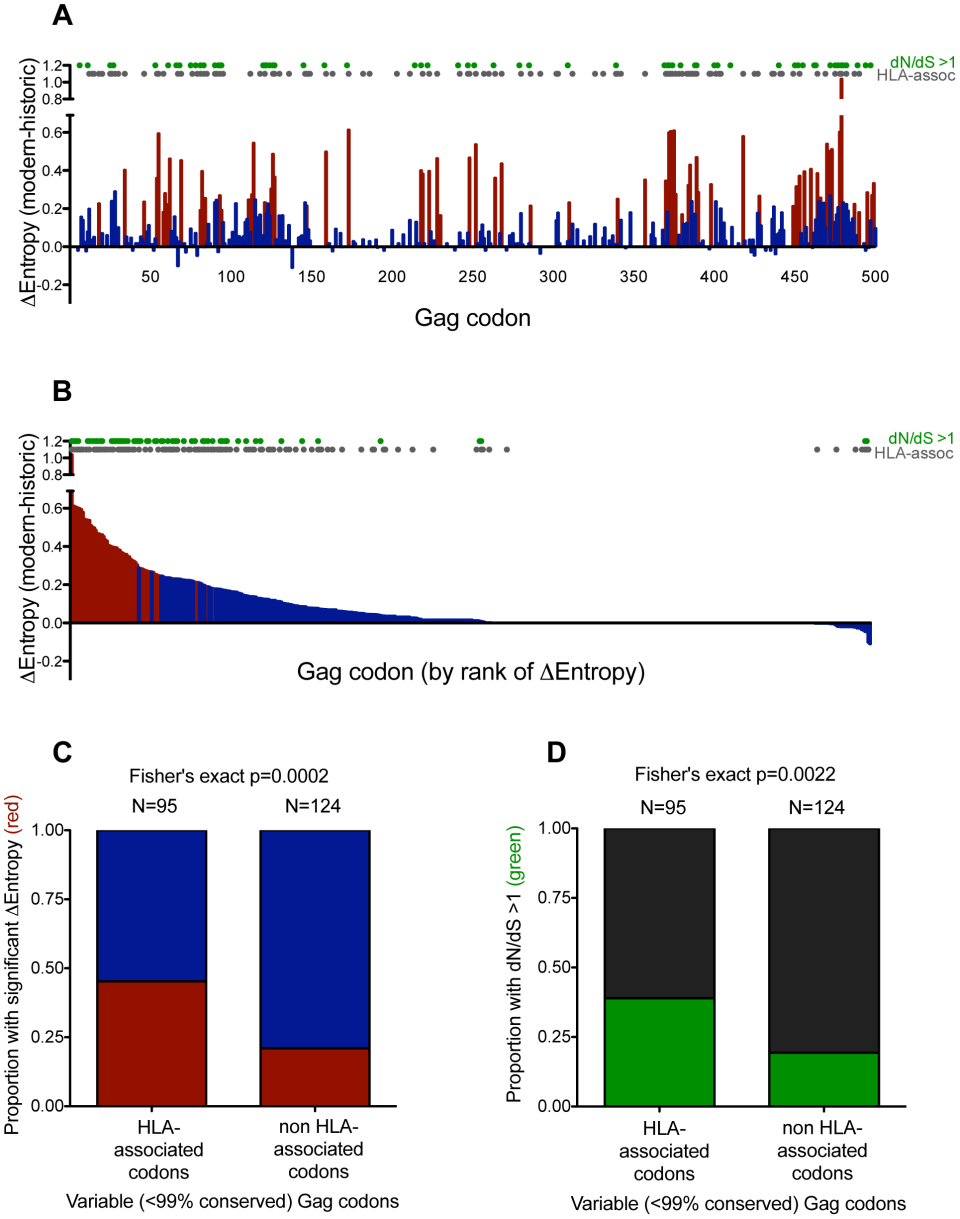
Gag residues exhibiting significant diversification over time are biased towards known HLA-associated sites. **Panel A:** Differences in Shannon entropy (Δentropy) between modern and historic sequences are shown for every Gag codon. Positive y-values indicate higher entropy in modern vs. historic sequences at that codon; negative y-values indicate the opposite. Red bars indicate significant entropy differences (defined as p<0.001, q<0.01); blue colors indicate differences that do not reach this significance threshold. Grey dots designate known HIV sites under selection by HLA (as defined in [43]). Green dots designate sites that display significant evidence of pervasive positive selection (dN/dS>1; posterior probability >0.9). **Panel B:** Same as panel A, but sorted by decreasing Δentropy rather than codon order. **Panel C:** Graphical depiction of a 2×2 contingency table stratifying variable (<99% conserved) Gag codons based on their status as HLA-associated (yes vs. no), and whether they exhibited significant Δentropy between modern and historic datasets (p<0.001 [red] vs. not [blue]). Ns are indicated above each bar. **Panel D:** Graphical depiction of a 2×2 contingency table stratifying variable (<99% conserved) Gag codons based on their status as HLA-associated (yes vs. no) and evidence that they are under significant pervasive positive selection (dN/dS>1; posterior probability >0.9 [green] vs. not [black]). Ns are indicated above each bar.

We began with Gag, by aligning historic and modern amino acid sequences to the HIV reference strain HXB2 and computing changes in Shannon Entropy on a per-codon basis (1000 bootstraps). This revealed 69 (of 500; 14%) codons whose entropies were significantly higher (p<0.001, q<0.01) in modern versus historic sequences (**Figures 3A, 3B**). To minimize circularity of arguments, we next excluded highly (>99%) conserved codons from consideration, as these cannot diversify to any great extent (and as such, are rarely identified as HLA-associated [43]) – leaving 219 “variable” Gag codons for analysis. Stratifying these sites by their HLA status indicated that, of the 95 Gag sites under selection by HLA [43], 45.2% exhibited significantly higher entropy in modern versus historic sequences, compared to 21.0% of the 124 sites not associated with HLA (p = 0.0002, **Figure 3C**). This indicates that HLA-associated viral sites tend to be those that have diversified the most between historic and modern-era HIV sequences.

While entropy approaches strictly investigate the end products of diversification, dN/dS-based approaches provide a more direct way to investigate elevated substitution rates within the phylogeny. As such, we identified sites under significant pervasive positive (diversifying) selection in a maximum-likelihood phylogeny comprising historic and modern sequences using the fast unconstrained Bayesian approximation for inferring selection algorithm [56]. As expected, after excluding codons that were >99% conserved, sites under pervasive positive selection were more likely to experience a significant increase in entropy (p<1×10^−5^, not shown) (indicating that positive selection is driving some of this diversification), and were more likely to be HLA-associated (suggesting that HLA represents a major source of this selection pressure) (p = 0.0022, **Figure 3D**).

We repeated these analyses for Nef, revealing trends consistent with those observed for Gag (**Figure S4**). Results thus suggest that ongoing HIV diversification is attributable, at least in part, to HLA pressures.

### Assessing the spread of HLA-associated polymorphisms in the population over time

We now turn to our major goal of assessing the spread of HLA-associated polymorphisms in the population over time. If escape mutations in HIV are reproducibly selected in individuals expressing particular host HLA(s), but such mutations consistently and rapidly reverted upon transmission, then we would expect their frequencies to be generally higher among individuals expressing the relevant HLA(s), and generally low among individuals *lacking* them, at levels that remain stable over time. But, if HLA-associated polymorphisms were to persist upon transmission and gradually spread in the population, we would expect polymorphism frequencies among HLA-matched individuals to remain stably higher, but polymorphism frequencies among individuals lacking the restricting HLA(s) to *increase* over time. As such, we stratified our HLA-associated polymorphism frequency comparisons between epidemic eras with respect to persons expressing, versus not expressing, the relevant HLA(s).

As before, we defined HLA-associated polymorphisms according to an independent source [43]. Because the present analysis investigated individual viral polymorphisms (rather than just sites) associated with HLA, a more specific definition was warranted. As such, we investigated all HLA-associated “adapted” (escape mutant) forms meeting a false-discovery rate threshold of <5% in the original study (see [43] and methods). This list comprised specific HLA-associated polymorphisms occurring at 71 Gag and 96 Nef codons [43]. HLA-associated polymorphisms in HIV were additionally stratified based on whether they represented consensus or non-consensus viral residues. Though the vast majority of HLA-associated polymorphisms represent non-consensus residues, a minority represent cases where an HLA allele is associated with preservation of the consensus residue at a given site (e.g. HLA-B*07:02 is associated with preservation of consensus G357 in Gag) [43]. We analyzed such cases separately because, under conditions of star-like diversification of a “consensus-like” founder, the null expectation is for polymorphism (*i.e.* non-consensus) frequencies to increase, and consensus frequencies to decrease, over time. Separating them also allows more intuitive interpretation when polymorphism frequencies are summarized as averages.

**Figure 4:**
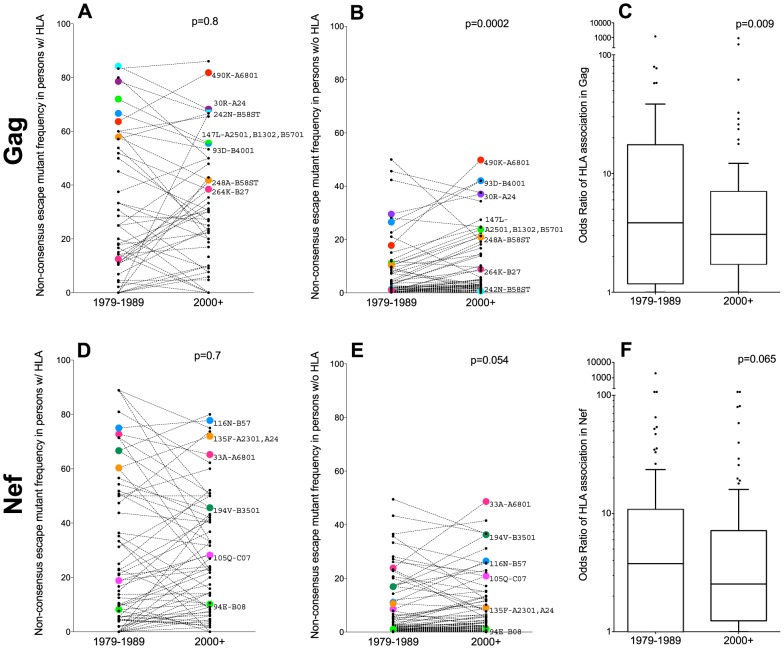
Differences in non-consensus escape mutant frequencies in persons expressing versus not expressing the restricting HLA allele(s), by era. **Panel A:** Frequencies of 70 published non-consensus HLA-associated polymorphisms (defined in [43]), in historic (1979–1989) and modern (2000+) HIV Gag sequences from individuals expressing the restricting HLA allele(s) are shown as linked pairs. A selection of well-known HLA-associated polymorphisms are labeled with their codons and restricting allele(s). P-values for all figure panels are computed using the Wilcoxon matched-pairs test. **Panel B:** Frequencies of these same 70 HLA-associated polymorphisms in historic and modern HIV Gag sequences from individuals *lacking* the restricting HLA allele(s). **Panel C:** Odds Ratios of association between these 70 HLA-associated Gag polymorphisms and their restricting HLA allele(s) in historic (1979–1989) and modern (2000+) cohorts. **Panel D:** Frequencies of 89 published nonconsensus HLA-associated polymorphisms in historic and modern HIV Nef sequences from individuals expressing the restricting HLA allele(s). **Panel E:** Frequencies of these same 89 HLA-associated polymorphisms in historic and modern HIV Nef sequences from individuals *lacking* the restricting HLA allele(s). **Panel F:** Odds Ratios of association between these 89 HLA-associated Nef polymorphisms and their restricting HLA allele(s) in historic and modern cohorts.

We began by investigating the frequencies of 70 non-consensus HLA-associated polymorphisms, occurring at 60 codons in Gag, between HLA-expressing and non-expressing persons in the historic and modern cohorts (**Figure 4**). As expected, individual polymorphism frequencies varied widely, but they were nevertheless enriched among individuals expressing the relevant HLA(s) (**Figure 4A**) compared to individuals lacking them (**Figure 4B**). In accordance with the null expectation, polymorphism frequencies in persons harboring the relevant HLA(s) were consistent across historic (median 18%, Interquartile Range [IQR] 4–54%) and modern (median 23% [IQR 7–45%]) cohorts (p = 0.8; **Figure 4A**). For example, Gag-242N frequency was ≥70% among persons expressing a B58 supertype allele, regardless of era. In persons lacking the relevant HLA(s), we also observed numerous examples of polymorphisms whose frequencies remained stable between historic and modern eras (*e.g.* Gag-242N frequency remained <1% in persons lacking a B58 supertype allele) (**Figure 4B**). Overall though, the average frequencies of these polymorphisms in persons lacking the relevant HLA(s) were modestly, yet statistically significantly, higher in modern (median 3.7% [IQR 2–19%]) compared to historic (median 2.0% [IQR 0.7–10%]) sequences (p = 0.0002; **Figure 4B**), a result consistent with the spread of many – though not all – HLA-driven polymorphisms in the population. Results remained significant after adjusting for minor inter-cohort differences in HLA frequencies (as these influence rates of polymorphism transmission) (p = 0.001, Wilcoxon one-sample test, not shown).

Under conditions where HLA-associated polymorphisms are, on average, slowly spreading through the population, we would expect the statistical associations between HIV polymorphisms and their restricting HLA(s) to concomitantly weaken. Indeed, this appeared to be the case. The median odds ratios of association between HIV Gag polymorphisms and their restricting HLA(s) were modestly lower in modern (median OR 3.1 [IQR 1.7–7.1]) compared to historic (median OR 3.8 [IQR 1.2–17.5]) cohorts (p = 0.009, **Figure 4C**).

Similar trends were observed for the 89 non-consensus HLA-associated polymorphisms occurring at 77 codons in Nef. Among persons expressing the relevant HLA(s), Nef polymorphism frequencies remained consistently elevated in historic (median 14% [IQR 3–50%]) and modern (median 15% [IQR 3–41%]) cohorts (p = 0.7; **Figure 4D**). In persons lacking the relevant HLA(s), examples of polymorphisms whose frequencies remained stable across historic and modern cohorts were noted (*e.g.* Nef-94E frequency remained ∼1% in persons lacking B*08, while Nef-135F remained ∼10% in persons lacking A*23:01 and A*24) (**Figure 4E**). Overall though, the average frequencies of these polymorphisms in persons lacking the relevant HLA(s) were modestly higher in modern (median 3.4% [IQR 1–12%]) compared to historic (median 2.0% [IQR 0.6–11%]) sequences, though this did not reach statistical significance (p = 0.054) (**Figure 4E**). Median odds ratios of association between Nef polymorphisms and their restricting HLA(s) were also slightly lower in modern (median 3.1 [IQR 1.7–7.1]) compared to historic (median 3.8 [IQR 1.2–17.5]) cohorts, though not significantly so (p = 0.065, **Figure 4F**).

We also investigated HLA-associated polymorphisms occurring at 11 Gag and 19 Nef codons where the association represented the consensus residue [43]. As expected, we observed higher frequencies of these consensus residues in individuals restricting the relevant HLA(s) compared to individuals lacking them (**Figure S5**). We also observed trends, though not statistically significant, towards lower consensus frequencies at these sites in modern versus historic sequences, regardless of HLA alleles expressed (**Figure S5**).

Taken together, our results are consistent with a scenario in which, on average, non-consensus HLA-associated polymorphisms have increased in frequency in North American HIV sequences over time. That said, the observed increases for Nef were not statistically significant, and both proteins harbored numerous examples of HLA-driven polymorphisms with stable background prevalence (*e.g.* Gag-242N, Nef-94E, Nef-135F). Moreover, although results for Gag attained statistical significance, average polymorphism background frequencies remained notably low, regardless of era. Our results thus indicate that not all HLA-driven polymorphisms are accumulating in circulation. Rather, our results suggest a diversity in accumulation rates, with the majority of nonconsensus polymorphisms spreading slowly (and others not at all) – and consensus residues decreasing in frequency overall. These observations confirm slow polymorphism spread predicted by mathematical models [9] and are consistent with an epidemic that is gradually diversifying under selection pressures that include HLA.

### Comparing the extent to which historic and modern sequences are “pre-adapted” to host HLA

Our results suggest that, on average, HLA-associated polymorphisms are spreading in the population, albeit slowly. From an immunological perspective, an increasing burden of escape mutations in circulating HIV strains over time could yield a reduction in the ability of individuals to control the virus via cellular responses as the epidemic progresses. We thus asked: if an individual were to be randomly infected by an HIV sequence from the historic or modern eras, to what extent would the latter contain a higher burden of polymorphisms that are “pre-adapted” to their HLA? To estimate this quantity, we compared each individual's HLA profile against all historic and modern chronic-phase HIV sequences in our dataset, and calculated the percentage of HLA-associated sites in each sequence exhibiting the adapted form specific to each person's total HLA profile. Comparison of the overall per-person averages thus represents the expected extent to which a randomly sampled HIV sequence would be pre-adapted to a given individual, had they been infected by a sequence from that era. Focusing first on non-consensus HLA-associated polymorphisms, our calculations for Gag yielded a median “percentage HIV sites pre-adapted to one's HLA profile” of 14.9% [IQR 10.1–19.5%] for historic versus a median of 17% [IQR 12.7–22.4%] for modern sequences, an average increase of only ∼2% (**Figure S6**). Inclusion of consensus HLA-associated polymorphisms further minimized this gap (not shown). For Nef, the median “percentage of adapted sites” remained consistent across eras (19.0% in historic versus 18.5% in modern) (**Figure S6**); moreover, inclusion of consensus polymorphisms resulted in lower overall percentages in modern compared to historic sequences (not shown). Results therefore suggest that, despite HIV diversification, an individual's overall expected risk of acquiring escape mutant viruses specific to their HLA allele profile has increased only minimally for Gag, and not at all for Nef, since the 1980s in North America.

### Polymorphisms restricted by protective HLA alleles appear to be accumulating to a greater relative (though not absolute) extent

Broadly speaking, at any given point in time, the average background frequencies of HLA-associated polymorphisms in circulating HIV sequences will generally positively correlate with the frequencies of their restricting HLA alleles in the population [12]. This is because higher absolute numbers of persons expressing the HLA will generally translate to higher absolute numbers of polymorphisms selected and thus transmitted (though many factors, including the wide-ranging probabilities of polymorphism selection given their location and restricting HLA, the fact that multiple HLA alleles select the same – or opposing – mutations at a given location, the existence of “consensus” HLA-associations, and the timing of polymorphism selection/reversion, will render this correlation less than perfect). Nevertheless, such a positive trend is observed in both the historic and modern cohorts, as expected (**Figure S7**). However, we are specifically interested in investigating the extent to which HLA-associated polymorphisms are spreading through the population over time. We thus asked: are polymorphisms restricted by certain HLA alleles increasing to a greater extent than others?

**Figure 5:**
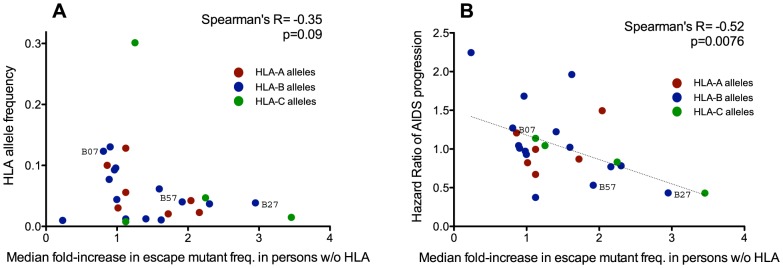
Protective HLA alleles are associated with the greatest relative increases in HLA-associated polymorphism background frequencies. Each dot illustrates a single HLA class I allele, colored red, blue and green, for HLA-A, -B, and -C alleles, respectively. **Panel A:** No significant correlation is observed between the frequency of a given HLA allele in the population (y-axis) and the relative extent to which its polymorphisms have spread over time (computed as the median fold-difference in background frequency of its associated polymorphisms in modern compared to historic HIV sequences; x-axis). This suggests that the accumulation of HLA-associated polymorphisms in circulating sequences is not simply driven by common HLA alleles. **Panel B:** A significant inverse correlation is observed between an HLA allele's Hazard Ratio of progression to AIDS ([57], y-axis) and the relative extent to which its polymorphisms have spread in the population over time (x-axis). This suggests that HLA-associated polymorphisms whose background frequencies have increased to the greatest relative extent between historic and modern eras are those restricted by protective HLA alleles.

To do this, we analyzed all HLA allele groups for which a minimum of three HLA-associated polymorphisms (regardless of whether they were consensus or non-consensus) were studied (25 alleles total). For each HLA-associated polymorphism, we computed its fold-increase in background frequency over time (for example, a hypothetical polymorphism with a background frequency of 1% in the historic cohort versus 2% in the modern cohort would equate to a two-fold increase). For each HLA allele we then calculated the median fold-increase in frequency of all polymorphisms restricted by it. Overall, we observed no significant correlation between the frequency of a restricting HLA allele and the relative extent to which its polymorphisms spread throughout the population between historic and modern cohorts (Spearman's R = −0.35, p = 0.09) (**Figure 5A**). Taken together with the results in **Figure S7**, this indicates that, at any given point in time, polymorphisms restricted by common HLA alleles will generally be found at higher absolute frequencies in a population than those restricted by rarer ones, but such polymorphisms do not appear to be spreading in the population to a greater relative extent (*i.e.* when expressed in terms of fold-change) over time.

Strong epidemiological links between host carriage of specific HLA class I alleles and HIV disease progression have been demonstrated in natural history studies (*e.g.*: [57]), with some alleles, notably HLA-B*57 and HLA-B*27, consistently associated with slower progression [57]–[59]. We therefore wished to investigate the relationship between an HLA allele's “protective” status (defined as its published Hazard Ratio for progression to AIDS [57]) and its median fold-increase in polymorphism background frequency between historic and modern eras. Of interest, we observed a significant inverse correlation between these two parameters (Spearman's R = −0.52, p = 0.0076) (**Figure 5B**), suggesting that polymorphisms restricted by protective HLA alleles have, in relative (fold-change) terms, spread to a greater extent in the population than those restricted by non-protective HLA alleles.

It is nevertheless important to contextualize these results in absolute terms. Of the six HLA-B*57-associated sites studied in Gag, historic sequences harbored a median 0 [IQR 0–1] B*57-associated polymorphisms at these sites, compared to 1 [IQR 0–2] in modern Gag sequences. Of the six B*57-associated sites in Nef (two of which represent “consensus” associations), both historic and modern sequences harbored a median of 2 [IQR 1–3] B*57-associated adapted polymorphisms. It thus remains unclear to what extent these modest absolute increases may compromise the protective effects of certain HLA alleles as the epidemic progresses.

### HLA-associated polymorphisms identified via association approaches are consistent between historic and modern cohorts

**Figure 6:**
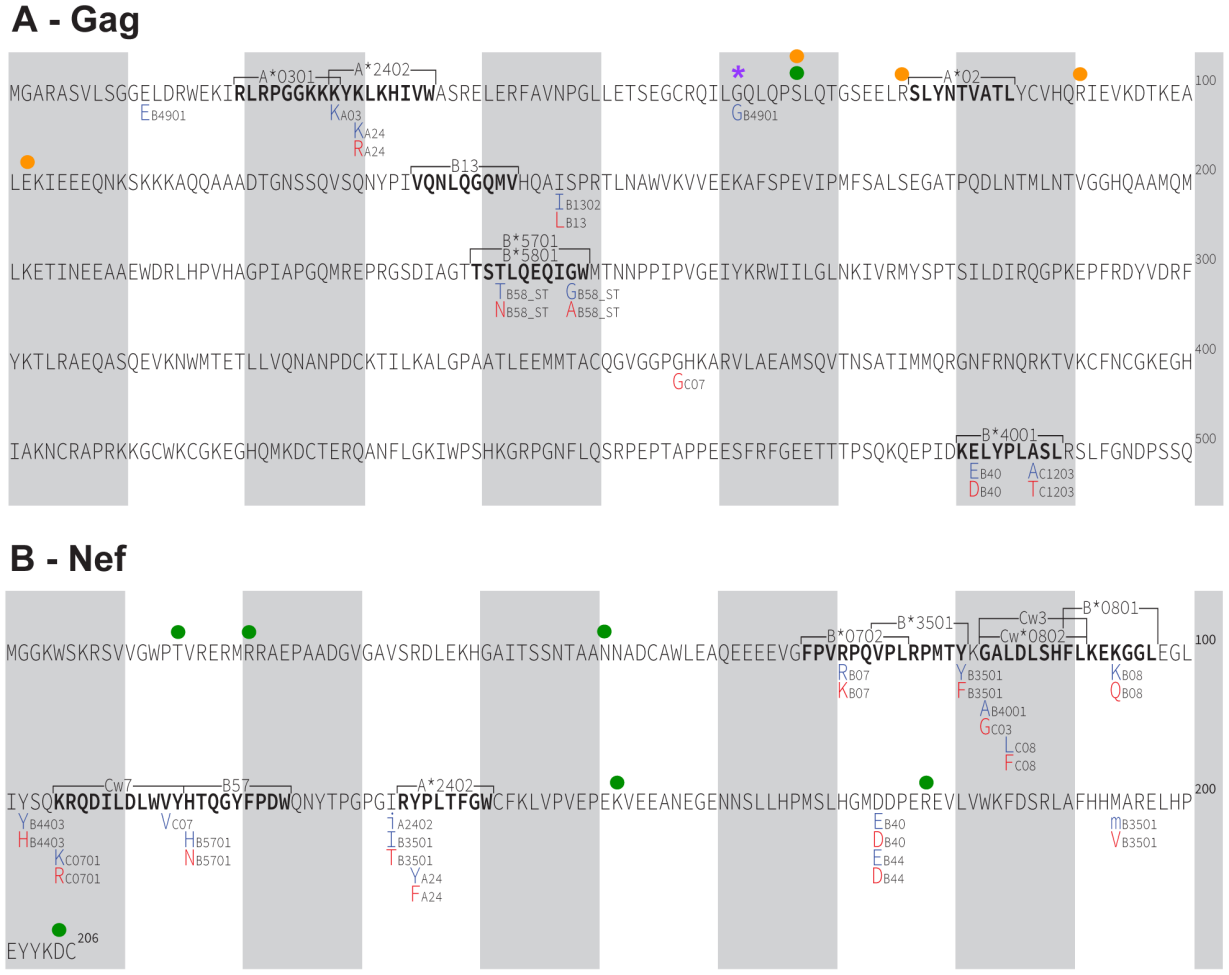
HLA-associated polymorphisms, identified via statistical association, in historic HIV sequences. **Panel A:** Gag immune escape map, indicating the locations, specific amino acid residues and HLA restrictions of HLA-associated polymorphisms identified at q≤0.05 in our historic cohort. The HIV consensus B amino acid sequence is used as a reference. Shaded vertical bars separate blocks of 10 amino acids. “Adapted” amino acids (those over-represented in the presence of the HLA allele) are red. “Nonadapted” amino acids (those under-represented in the presence of the HLA allele) are blue. UPPERCASE letters distinguish polymorphisms that survive correction for HIV codon covariation (“direct” associations), while lowercase letters distinguish polymorphisms that do not survive correction for codon covariation (“indirect” associations). The notation “_ST” following an HLA (*e.g.* B58_ST) identifies associations identified at the supertype level. The locations of optimally-defined, HLA-restricted CTL epitopes straddling or adjacent to HLA-associated polymorphisms are indicated. The well-known A*02-SL9 epitope (SLYNTVATL) epitope is also shown; no historic HLA-associated polymorphisms were identified therein at q<0.05. The single “novel” historic HLA-associated polymorphism (B*49:01-62G) is indicated with a purple asterisk. A green filled circle denotes the single Gag residue (codon 67) where the ancestral founder sequence was reconstructed with <80% confidence. Orange filled circles denote the four Gag residues (67, 76, 91 and 102) where the inferred ancestral founder sequence differs from the published North American subtype B consensus sequence. None of these sites harbor HLA associations. **Panel B:** Nef historic immune escape map. Green filled circles denote the six Nef residues where the ancestral founder sequence was reconstructed with <80% confidence (15, 21, 51, 152, 178, 205); none harbor HLA associations.

We have thus far defined HLA-associated polymorphisms as those identified in independent modern cohorts by statistical association [43]. To investigate the potential existence of novel historic HLA-associated polymorphisms that are no longer detectable in modern sequences due to their spread throughout the population, we applied association testing approaches to our historic dataset directly. Historic patients with known or suspected early infection were excluded (as these could dilute associations between HLA and HIV polymorphisms due to insufficient within-host evolution), and a false-discovery rate (q-value) cutoff of 0.05 was employed. We were especially interested to see whether HIV codons whose inferred ancestral (founder) amino acid differed from the North American consensus (there were 4 in Gag) or were reconstructed with <80% confidence (1 in Gag and 6 in Nef) could be explained by the existence of historic HLA-associated polymorphisms at these sites. However, no such evidence was observed (**Figure 6A, 6B**). Instead, analysis revealed 16 HLA-associated polymorphisms occurring at 10 Gag codons and 28 HLA-associated polymorphisms occurring at 13 Nef codons that, with the exception of an association between B*49:01 and the consensus G at Gag codon 62, were wholly consistent with published escape pathways [**43**] and/or were confirmed in the present modern cohort (not shown). In summary, the strongest HLA-associated polymorphisms in historic sequences are consistent with those identifiable today.

### Gag and Nef function of ancestral, historic and modern HIV sequences

**Figure 7:**
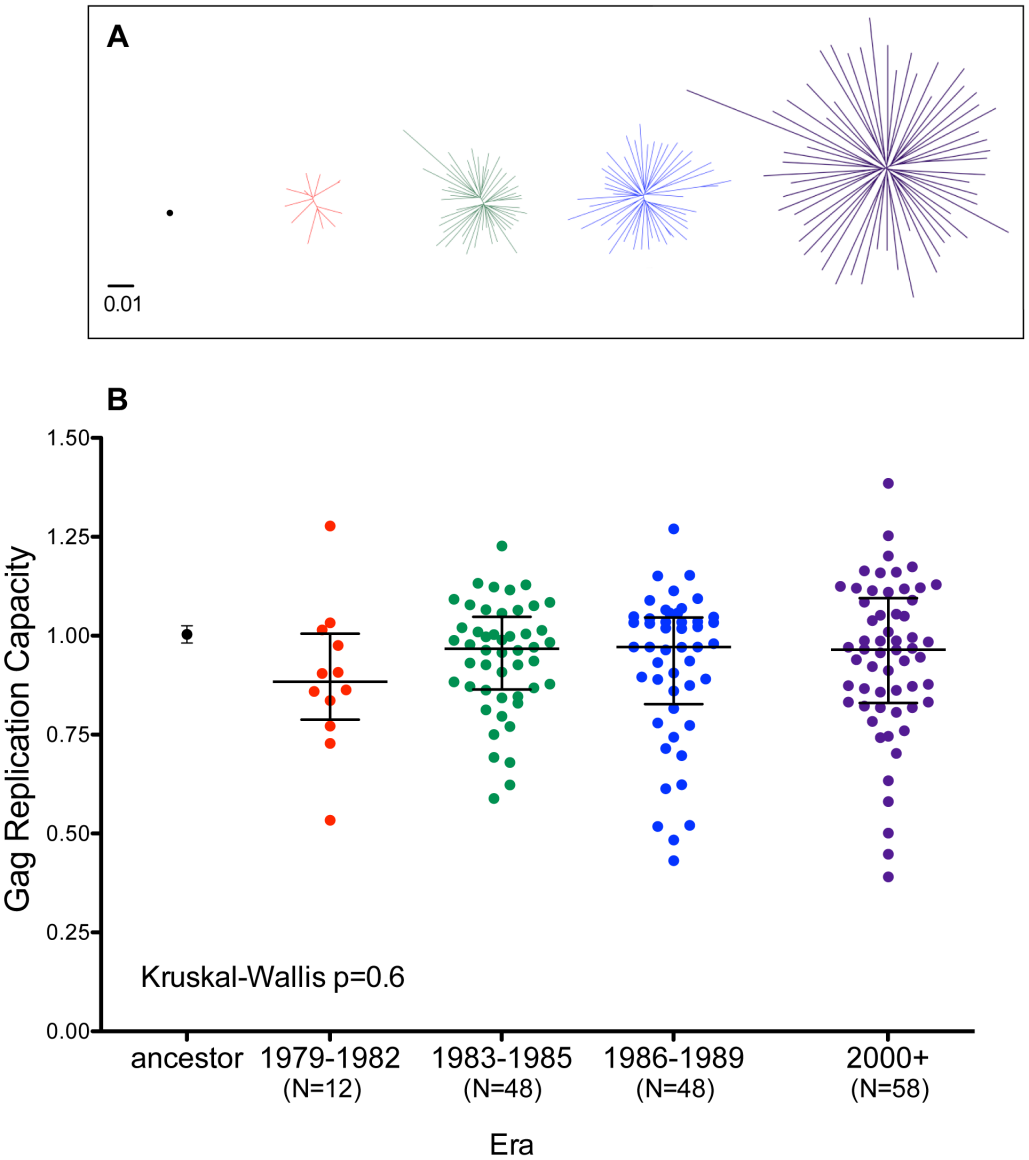
Replicative implications of Gag diversification during the North American Epidemic. **Panel A:** Unrooted Maximum-Likelihood phylogenies, drawn on the same distance scale, depicting the inferred ancestor (single black dot), early-historic (red, 1979–1982), mid-historic (green, 1983–1985), late-historic (blue, 1986–1989) and modern (purple, 2000+) Gag clonal sequences from unique patients that were used to construct recombinant NL4-3 viruses for functional assessment. **Panel B:** NL4-3 normalized replication capacities of recombinant viruses containing the Gag sequence of the inferred ancestral sequence (Mean±S.E.M. of 3 replicate measurements) as well as patient-derived Gag clonal sequences (one per patient, representing the mean of ≥2 replicate measurements). An RC of 1 indicates replication equal to that of NL4-3 while RC>1 and <1 indicate RC higher or lower than NL4-3 respectively. Although visually there appears a trend towards lower replication capacity among Gag clones from early historic (1979–1982) era, there no significant differences in RC between any of the groups (Kruskal-Wallis test, p = 0.6).

HIV Gag and Nef are highly immunogenic HIV proteins whose sequence variability is substantially influenced by HLA [43] and whose function is susceptible to immune-mediated attenuation [44]–[46]. As such, we investigated whether the gradual spread of immune escape mutations in North American Gag and Nef sequences may be accompanied by overall changes in the average viral replication capacity and/or protein function of patient-derived HIV sequences. We began with Gag, by generating a recombinant HIV strain expressing the epidemic's inferred Gag ancestral sequence, and another expressing the published global subtype B consensus (**Figure S3**) in an HIV NL4-3 subtype B reference strain backbone. We also generated recombinant HIV NL4-3 strains expressing a single representative clonal Gag sequence from 108 (of 120 originally selected; 90.0% success rate) historic and 58 (of 71 originally selected; 82% success rate) modern specimens (**Figure 7A**). A clonal (rather than quasispecies [60]) approach was adopted for the patient-derived sequences, as variations in viral stock diversity resulting from differential integrity of historic versus modern specimens could bias replicative measurements. We assayed the *in vitro* replication capacity of these recombinant viruses using a published reporter T-cell assay [60]–[63]. Replication capacities (RC) were normalized to that of parental NL4-3, such that values >1 and <1 indicate RC greater or less than NL4-3, respectively.

The replication capacities of recombinant viruses encoding the inferred ancestral and global subtype B consensus sequences were comparable to those of parental NL4-3 (**Figures 7B** **and S8**). Recombinant viruses expressing historic or modern Gag clonal sequences displayed a broad range of growth phenotypes, with median RCs approaching that of NL4-3 (**Figure 7B**). Although there appeared to be a trend towards lower RC among Gag recombinant viruses from early historic (1979–1982) patients, this was not statistically significant (Kruskal-Wallis p = 0.6). Furthermore, no correlation was observed between the replication capacity of a given Gag clone and its genetic distance from the Gag NL4-3 sequence (Spearman's R = 0.03, p = 0.6, not shown), arguing against confounding effects attributable to our use of a historic lab-adapted sequence (NL4-3) as a viral backbone.

**Figure 8:**
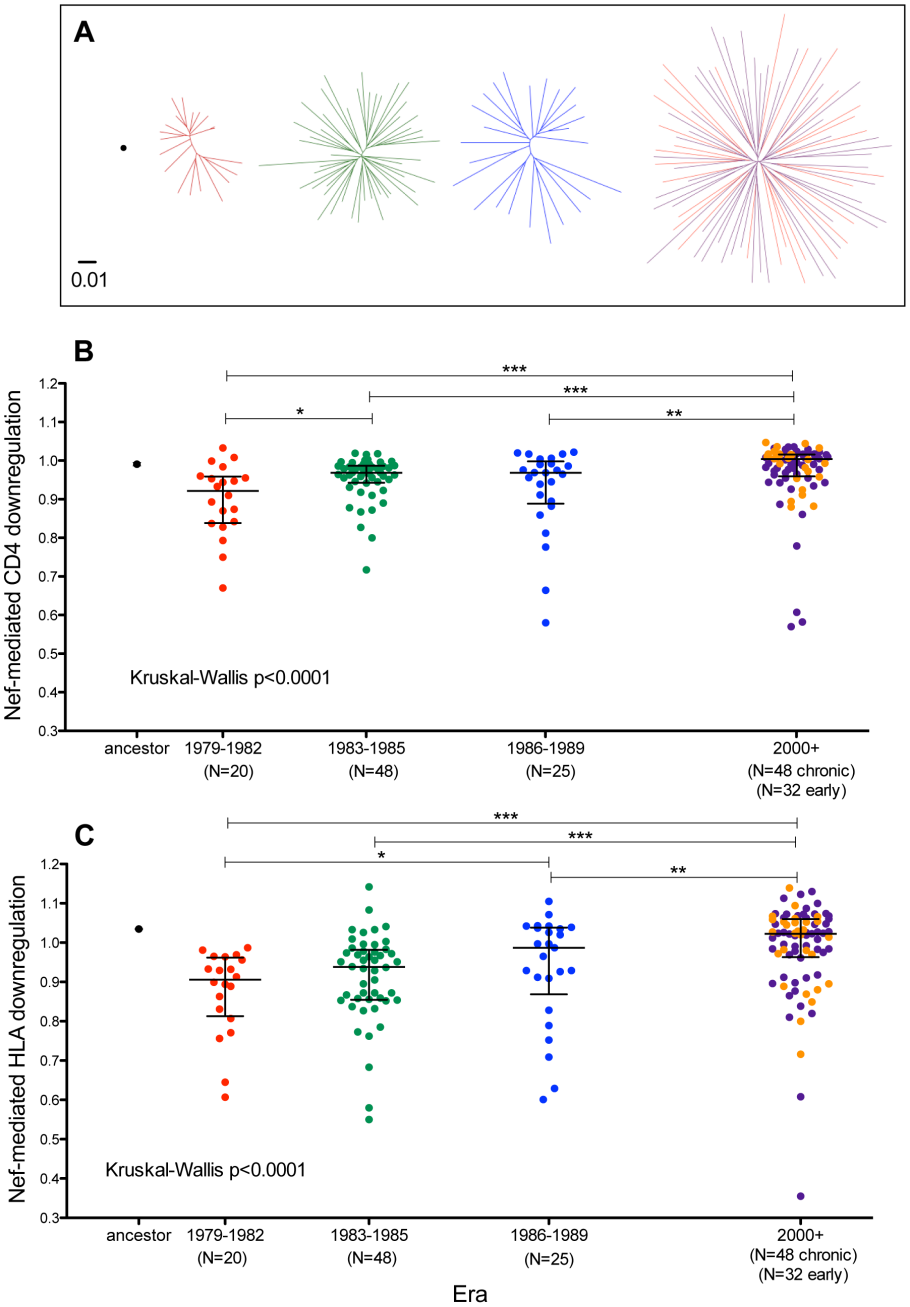
Functional implications of Nef diversification during the North American Epidemic. **Panel A:** Unrooted Maximum-Likelihood phylogenies, drawn on the same distance scale, depicting the inferred ancestor (single black dot), early-historic (red, 1979–1982), mid-historic (green, 1983–1985), late-historic (blue, 1986–1989) and modern (purple: chronic-phase, orange: acute-phase, year 2000+) Nef clonal sequences from unique patients cloned into a GFP-expression vector for functional assessment. **Panel B:** CD4 downregulation activities of the inferred ancestral Nef sequence (mean±S.E.M. of 8 replicate measurements) and patient-derived Nef clones from various eras (one per patient, representing the mean of triplicate measurements). CD4 downregulation values are normalized to that of HIV subtype B control Nef strain SF2, such that a value of 1 indicates CD4 downregulation activity equal to that of SF2 while values>1 and <1 indicate activities higher or lower than SF2 respectively. Modern Nefs exhibited significantly higher CD4 downregulation activity compared to historic Nefs (Kruskal-Wallis p<0.0001). **Panel C:** SF2-normalized HLA class I downregulation activities of inferred ancestral (mean±S.E.M. of 8 replicate measurements) and patient-derived Nef sequences (one per patient, mean of triplicate measurements). Modern Nefs exhibited significantly higher HLA downregulation activity compared to historic Nefs (Kruskal-Wallis p<0.0001).

Similarly, we cloned the inferred ancestral, global subtype B consensus and a single representative Nef sequence from N = 102 historic and N = 86 modern patients into a GFP-expression vector (**Figures 8A** **and S8**). As modulation of Nef function over the natural history of infection is supported by some [64], [65] (though not all [66]) studies, and a minority of historic Nef clones were derived from persons with known or suspected early infection, we indirectly assessed infection stage as a potential confounder by including Nef sequences from 52 modern chronic and 34 early infection patients not included in previous analyses (sampled a median of 72 [IQR 48–92] days after infection) in our comparison group. Following transient transfection into an immortalized T-cell line stably expressing CD4 and HLA-A*02, we assessed the ability of these Nef clones to downregulate these molecules from the cell surface by flow cytometry [67], [68] (**Figure 8B**). The Nef sequence from HIV reference strain SF2 served as a positive control (SF2 is commonly used as a control in Nef functional studies, as it possesses robust CD4 and HLA class I downregulation activities, *e.g.* [67]); thus, normalized Nef functions of >1 and <1 indicate activity greater or less than SF2, respectively. Nef protein expression was verified by Western blot (**Figure S8**); 15 poorly functional Nef clones whose expression could not be detected were excluded (since *in vitro* cloning defects or other artifacts could not be ruled out), leaving 93 historic and 80 modern clones for analysis.

CD4 downregulation activity of ancestral Nef was comparable to that of reference strain SF2 (**Figure 8B**), while that of global subtype B consensus Nef was ∼3% lower (not shown). Nef clones from historic and modern patients were generally highly functional for CD4 downregulation and exhibited relatively narrow dynamic ranges. Nevertheless, historic patient-derived Nef sequences exhibited significantly lower CD4 downregulation abilities compared to modern sequences (Kruskal-Wallis p<0.0001), with the early (1979–1982) Nef clones exhibiting the lowest function overall (**Figure 8B**). Nef-mediated CD4 downregulation of modern Nef clones from individuals in early and chronic infection were comparable (p = 0.9, **Figure 8B** and not shown), arguing against infection stage as a major confounder of this result.

The ability of the ancestral Nef sequence to downregulate HLA-A*02 was ∼3.5% higher than reference strain SF2 (**Figure 8C**), while that of global subtype B consensus Nef was equivalent to SF2 (not shown). Although Nef clones from both historic and modern patients were in general highly functional, historic Nef sequences exhibited significantly lower HLA downregulation abilities compared to modern Nef sequences (Kruskal-Wallis p<0.0001), with the early (1979–1982) Nef clones displaying the lowest function overall (**Figure 8C**). HLA downregulation capacities of modern early Nef sequences were on average 1% higher than those from modern chronic Nef sequences (p = 0.14, **Figure 8C** and not shown), arguing against infection stage as a major confounder. The significantly lower Nef-mediated CD4 and HLA downregulation observed in historic versus modern sequences was robust to inclusion/exclusion of the 15 clones whose Nef expression was not detectable by Western Blot (not shown).

Taken together, the lack of significant functional differences between ancestral, subtype B consensus, and median patient-derived Gag clones from historic and modern eras argues against major replicative consequences of HIV Gag diversification during the North American epidemic. In contrast, our Nef results suggest the introduction of a highly functional founder virus to North America in the 1960s, followed by a subsequent decline in average Nef-mediated CD4 and HLA downregulation functions of patient-derived sequences in the 1980s, that were restored to original (“founder”) levels by the 2000s. The mechanisms and potential role for host pressures in this phenomenon require further investigation.

## Discussion

The present study examined linked host (HLA) and HIV (Gag/Nef) datasets from historic (1979–1989) and modern (2000–2011) eras in North America to estimate the extent to which HLA-driven polymorphisms may be spreading throughout circulating HIV sequences over time on this continent. Phylogenies inferred from historic and modern samples of HIV Gag and Nef sequence variation were star-like in shape, yielding a reconstructed ancestral (epidemic founder) virus sequence that was essentially identical to North American subtype B consensus. Mean pairwise distances between modern HIV Gag and Nef sequences were approximately two-fold greater than those between historic sequences, supporting a diversifying epidemic. Notably, Gag and Nef codons exhibiting the most significant entropy increases over time were enriched for known HLA-associated sites, consistent with a key role of HLA in driving HIV diversification [69], [70].

Also consistent with an approximate two-fold increase in HIV diversity since the mid-1980s in North America, the average “background” frequencies of HLA-associated polymorphisms (*i.e.* in individuals *lacking* the restricting HLA) were roughly two-fold higher in modern compared to historic sequences. These differences reached statistical significance for Gag, though not for Nef. As expected, in both historic and modern cohorts, a general positive correlation was observed between the frequency of an HLA allele and the background frequency of its associated polymorphism in the general population. However, the polymorphisms that, over time, appeared to be spreading to the greatest relative extent (in terms of fold-change) were not those restricted by common HLA alleles (**Figure 5A**) but rather those restricted by protective HLA alleles [57] (**Figure 5B**). This observation, along with our lack of identification of novel historic HLA-associated polymorphisms restricted by common HLA alleles, indicates that HIV is not simply adapting to the most frequent HLA alleles in a given host population. Instead, our findings are consistent with protective HLA alleles as those imposing the strongest evolutionary pressures on HIV, an observation that is consistent with previous reports that protective HLA alleles are more likely to induce strong selection at key conserved sites [43], [71]–[73].

The spread of HLA-associated polymorphisms in circulation could lead to a reduction in host antiviral immune potential over time [12]. We thus wished to interpret our results in terms of the imminence of this potential outcome. First and notably, the extent of HLA-driven polymorphism accumulation in Nef did not reach statistical significance. Second, though observations for Gag *did* achieve significance, average polymorphism background frequencies remained low in absolute terms (*i.e.* 2.0% in the 1980s versus 3.7% in the 2000s) – differences that, when expressed in terms of the average estimated extent to which circulating HIV Gag sequences are “pre-adapted” to an individual's HLA profile, translated into an overall increase of only ∼2% between historic and modern eras. Moreover, we observed numerous HLA-associated polymorphisms whose prevalence remained stable in the population (*e.g.* B58-supertype-associated Gag-242N, B*08-associated Nef-94E, A*2301/A*24–associated Nef-135F), observations that are consistent with their rapid reversion upon transmission [5], [9], [74] (though estimates of the reversion rate for B*08-Nef-94E are somewhat conflicting [9], [74]). That some - though certainly not all - HLA-driven escape mutations are capable of spreading through the population has been demonstrated via mathematical modeling [9], indicating that the reproducible selection of specific escape mutations in persons harboring the relevant HLA does not always translate into rapid evolution at the population level [9]. That certain HIV sites simultaneously display strong signals for diversifying selection, yet stable polymorphism prevalence, is also consistent with “toggling” between consensus and escape forms [75] as HIV disseminates in a genetically diverse host population.

Although our study did not formally attempt to model the dynamics of HLA-driven polymorphism spread in the North American population, our observations suggest that this is happening slowly. Very gradual polymorphism spread is also consistent with mathematical models projecting that, even in the case where an escape mutation never reverts, it could take centuries for it to reach fixation following its initial appearance in the population [9]. Moreover, it has been projected that any reversion (however slow) would prevent a polymorphism from ever becoming fixed [9]. Also consistent with slow spread is the near-identity of the reconstructed epidemic MRCA (founder) HIV sequence to the North American consensus - which suggests that, between the North American epidemic's genesis and the present day, no polymorphism, HLA-driven or otherwise, has spread to an extent where it now outcompetes that of the original founder residue. Our lack of identification of novel historic HLA-associated polymorphisms at the seven Gag/Nef codons where the inferred ancestor was reconstructed with <80% confidence and the four (highly variable) Gag codons where it differed from the modern consensus also argues against the spread of any historic HIV escape mutation in North America to the point where it now defines consensus. Note however that some caution is merited when interpreting the estimated founder viral sequence, since rapid selective sweeps occurring between the epidemic's foundation [54], [55] and the earliest 1979 sampling date would not have been detected and therefore cannot be ruled out. Acknowledging these caveats, the near-identity between the estimated North American founder virus and modern consensus additionally suggests that statistical associations between particular HLA alleles and the HIV consensus residue at a given site (*e.g.* B*07:02 with Gag-G357) have *not* arisen as a result of their selection and subsequent spread in the population to the point where they define the consensus [10]. Rather, these residues were most likely present at the epidemic's foundation - and, if anything, are gradually *decreasing* in frequency as HIV continues to diversify. We propose that such “consensus HLA associations” represent cases where the founder virus happened to be adapted to certain HLAs (perhaps because the original founder or earlier hosts expressed them), and that these HLAs continue to exert purifying selection on these sites over time.

Despite inferred overall slow rates of accumulation, the observation that polymorphisms restricted by protective alleles appear to be spreading to a greater (relative) extent than others is potentially important. Indeed, the stabilization of certain protective allele-associated escape mutations by secondary (compensatory) substitutions has been documented: the S173A mutation (which allows the B*27-associated Gag-R264K mutation to persist upon transmission in an HIV subtype B context [11], [13]) and the S165N mutation (which stabilizes B*57-associated mutations within the p24^Gag^ KF11 epitope in a subtype C context [8]), are examples.

Despite this, we urge caution in extrapolating that the protective effects of HLA alleles will diminish rapidly in North America. Again, it is important to consider that absolute polymorphism background frequencies remain low: modern Gag and Nef sequences together harbor, on average, only one additional B*57-associated polymorphism compared to historic sequences. Similarly, despite polymorphism spread, a B*27-expressing individual still has a >90% chance of acquiring HIV with the immunologically susceptible consensus R at critical Gag codon 264. Besides, the protective effects of most such alleles (including, to a certain extent, B*27 [76]) are attributable to consistent and strong CTL responses against multiple HIV epitopes [43], [77], [78]. It is also important to consider that protective HLA-restricted CTL retain activity against polymorphic variants in many cases [79], [80], and *de novo* [81] or cross-reactive [82] CTL responses to *in vivo* escape variants can, and do, arise. Further integrated evolutionary and molecular studies are therefore required to assess the potential immunologic impact of polymorphism spread on HIV control by protective HLA alleles.

Our study also investigated whether HIV evolution in North America has been accompanied by changes in viral replication capacity or protein function. Consistent with previous *in vitro* assessments of HIV sequences reconstructed using Center-of-Tree approaches [83], our inferred Gag and Nef ancestral sequences were highly functional. Despite substantial increases in Gag diversity over time, the average replication capacities of recombinant NL4-3 viruses expressing patient-derived clonal Gag sequences from historic and modern eras were comparable to that of NL4-3 expressing the inferred Gag ancestral sequence, arguing against major replicative consequences of HIV Gag diversification during the North American epidemic. These results contrast with reductions in replication capacity of recombinant viruses expressing patient-derived Gag-protease sequences from Japanese patients from the mid-1990s to present [32], a difference possibly due to the greater homogeneity of HLA alleles in Japanese compared to North American populations, that may exert consistent selection pressures driving the selection of fitness-reducing mutations.

In contrast, the average Nef-mediated CD4 and HLA downregulation activities of historic patient-derived sequences were modestly yet significantly lower than modern ones. This is intriguing since the inferred Nef ancestral sequence displayed high function. We therefore speculate that, following the introduction of a functional ancestral Nef sequence into North America, initial HIV adaptation to this new population led to decreases in Nef function that were subsequently rescued upon continued Nef diversification. The higher Nef-mediated HLA class I downregulation function of modern compared to historic sequences, combined with the observation of modest HLA-driven polymorphism spread through the population during this same period, raises the interesting possibility that, compared to viruses circulating in the 1980s, modern North American HIV sequences may exhibit greater immune evasion potential via enhanced HLA class I downregulation [84] function. However, further studies will be required to elucidate the underlying mechanisms and pathogenic implications of these observations.

An anticipated criticism is our definition of HLA-associated polymorphisms by statistical association studies of modern cohorts [43]. This approach could underestimate the average extent of polymorphism spread over time, for two reasons. First, such lists could exclude historic escape mutations that are no longer detectable in modern cohorts due to polymorphism spread. To address this we applied statistical association approaches to identify HLA-associated polymorphisms detectable at the population level in the historic cohort. However, all identified polymorphisms save one were consistent with known HLA-associated escape pathways, indicating that the strongest mutations detectable historically remain readily detectable today. A second limitation is that association testing approaches, even those that incorporate phylogenetic correction (as ours do), systematically favor the identification of HLA-associated mutations that escape and revert rapidly [85], which by definition would not be expected to spread quickly in a population [9]. However, this limitation is somewhat offset by the substantial size (N>1800) of the cohort used to define HLA associations. Mathematical models indicate that at such sample sizes, with phylogenetic correction, significant associations can be detected between HLA alleles and polymorphisms even if these escape and/or revert on a timescale of decades [85]. Moreover we have previously demonstrated that cohorts of this size are powered to detect very rare HLA-associated polymorphisms, as well as those that are nearly universally observed in the population [43].

This study possesses additional limitations, many inherent to convenience sampling and technical challenges of working with historic samples. Although our sequences date back to 1979, the lack of data from the critical period between HIV's introduction into North America and the late 1970s is a major limitation of this and all other studies undertaken to date. Nevertheless, our historic HIV sequence dataset is 10-fold (Gag) and 7-fold (Nef) larger than existing data from this era and region, and includes the oldest North American sequences ever published. Another limitation is that specimens were obtained from only four sites in North America, and all historic specimens were derived from observational studies of individuals from a single risk group (MSM) [34], [36]–[42]. As such, our HIV diversity estimates, particularly for the historic era, may represent underestimates. Nevertheless, the dispersion of published North American HIV sequences throughout all phylogenies, the consistency of historic and modern consensus sequences, and our estimated epidemic founder dates that are compatible with published estimates [53]–[55] suggest that our sequences are not grossly unrepresentative of the North American epidemic. Concerns regarding our ability to faithfully amplify the original quasispecies diversity from historic specimens by PCR led us to adopt a single representative clone (rather than bulk) approach for our functional assessments of Gag and Nef in order to minimize *in vitro* bias associated with differences in the diversity of viral stocks. The presence of individuals with known or presumed early infection in our historic cohort and the general lack of clinical staging information are also limitations. To reduce confounding, early sequences were excluded from relevant analyses (*e.g.* identification of HLA-associated polymorphisms in the historic cohort and calculation of Odds Ratios of association between HLA and polymorphisms), while other analyses verified the appropriateness of pooling data by comparing early and chronic sequences directly to rule out differences between them (*e.g.* Nef functional assessments). The absence of pVL and CD4 information on historic patients also precluded the investigation of trends in disease markers over time.

On the other hand, our development of a sensitive HLA sequence-based typing assay capable of utilizing genomic DNA extracted from plasma/serum [48] allowed us to perform HLA typing of historic specimens, yielding, for the first time, the ability to directly investigate HLA-associated selection pressures over the course of an epidemic. A known limitation of serum-based HLA typing is the overrepresentation of homozygous types due to amplification bias [48], an effect that was noted in our historic dataset. Though this could lead us to overestimate the historic background frequencies of HLA-associated polymorphisms by erroneously including individuals expressing the relevant HLA into our calculations, the low average background frequencies of HLA-associated polymorphisms in modern sequences indicate that any overestimations would not substantially impact our overall conclusions. A notable strength is the lack of overlap between study cohorts and those from which the reference list of HLA-associated polymorphisms was derived [43], thus ensuring independence of source and query data.

In conclusion, HLA-associated polymorphisms are, on average, slowly spreading throughout North American HIV sequences as the epidemic continues to diversify. This slow adaptation to host cellular immune responses parallels the observed drift of HIV towards a more neutralization-resistant phenotype as a result of population-level viral adaptation to humoral immune pressures [86], [87]. However, the absolute frequencies of these polymorphisms in circulation remain on average low on this continent, as do the estimated risks of acquiring HIV “pre-adapted” to one's HLA profile. As such, our results are unlikely to translate into major imminent consequences to CTL-mediated control of HIV, at least in the North American region.

That said, we acknowledge that even modest changes can have biological implications. Indeed, one could contend that modest increases in the frequency of “pre-adapted” HIV strains are not inconsistent with reports suggesting increased HIV virulence over time [22]. Furthermore, it is important to emphasize that the potential rates, and thus immunologic implications, of HLA-associated polymorphism spread may be substantially greater in populations where HLA diversity is far lower and/or HIV prevalence far higher than North America. Rates and implications of polymorphism spread may also be more profound in populations where transmission tends to occur later in infection, thereby increasing the probability of transmitted escape mutations (though mathematical models have suggested that realistic differential transmission rates between acute and chronic infection would impact population escape mutation prevalence only minimally [9]). As such, we recommend that similar analyses of virus-host adaptation be undertaken to assess the rate of accumulation of immune-driven polymorphisms, and its pathogenic implications, in other epidemic regions where historic specimens are available. In conclusion, though our results remain somewhat open to interpretation, we suggest that they be considered in light of the major advances in HIV treatment and prevention [88]–[92] that have occurred during the timecourse of the present study. Combined with current efforts in prevention and cure research [93]–[95], these advances give us firm hope that the end of HIV/AIDS will precede the virus' ability to fully subvert host cellular immunity through population-level adaptation.

## Methods

### Ethics statement

Research subjects, all adults, were enrolled under REB-approved protocols and provided written informed consent to participate in the original studies for which specimens were collected. Ethical approval to conduct this study was obtained from the Institutional Review Boards at Providence Health Care/University of British Columbia and Simon Fraser University.

### Historic and modern cohorts

A total of 497 historic plasma/serum specimens from unique patients enrolled in observational studies of men who have sex with men (MSM) at four North American sites between 1979–1989, were obtained for study. Of these, 94 and 75 were from the New York Blood Center (NYBC; 1979–1989) and the San Francisco Department of Public Health (SFDPH; 1979–1984), respectively, and represented participants of hepatitis B observational studies whose archived sera were retrospectively tested for HIV [34], [38], [39]. A further 282 and 46 were obtained from the Fenway Community Health Clinic in Boston (Fenway; 1985–1989) [36], [37] and the Vancouver Lymphadenopathy-AIDS Study in Vancouver, Canada (VLAS; 1984–1987) [40]–[42]. With the exception of 67 NYBC patients whose dates of HIV infection were estimated to be within 6 months prior to specimen collection, all other patients were known or presumed to be in chronic infection. Specimen integrity varied by cohort. Whereas sera from NYBC, SFDPH and Fenway were stored at −70°C since collection, VLAS specimens had been stored at −20°C and bore evidence of freeze-thaw cycles. No clinical information (*i.e.* plasma viral load, CD4) was available for historic specimens; furthermore, sociodemographic and other identifying information were not sought. Our modern comparison cohort comprised 382 individuals for whom HIV Gag and/or Nef sequences were available: 26 were recruited through the Aaron Diamond AIDS Research Center in New York, 91 from Massachusetts General Hospital in Boston and 265 from various cohort studies based at the BC Centre for Excellence in HIV/AIDS in Vancouver, Canada. The modern cohort comprised MSM, injection drug users and individuals with unknown HIV risk group.

### Viral and host genotyping

HIV RNA was extracted from plasma or serum using standard methods. Gag and Nef regions were amplified by nested RT-PCR using sequence-specific primers and amplicons were bidirectionally sequenced on a 3130xl and/or 3730xl automated DNA sequencer (Applied Biosystems). Data were analyzed using Sequencher v5.0 (Genecodes) or RECall [96] with nucleotide mixtures called if the height of the secondary peak exceeded 25% of the height of the dominant peak (Sequencher) or 20% of the dominant peak area (RECall). All HIV sequences were confirmed as subtype B using the recombinant identification program (RIP; http://www.hiv.lanl.gov/content/sequence/RIP/RIP.html). HXB2-alignments were performed using an in-house tool based on the HyPhy platform [97]. Phylogenetic trees were constructed using maximum-likelihood approaches [98] and visualized using FigTree (http://tree.bio.ed.ac.uk/software/figtree/). Patristic (pairwise) genetic distances were computed using PATRISTIC [99]. Intercohort comparisons of Shannon entropy scores (featuring 1000 randomizations with replacement) were performed using Entropy-two (http://www.hiv.lanl.gov/content/sequence/ENTROPY/entropy.html). Detection of HIV Gag and Nef codons exhibiting significant evidence of pervasive positive selection (defined as having a posterior probability ≥0.9 that the site-specific nonsynonymous rate exceeds its synonymous rate) in the combined historic/modern datasets was performed using the fast unconstrained Bayesian approximation for inferring selection algorithm [56], implemented in Datamonkey [100], [101].

Consensus sequences were calculated by plurality rule. North American Gag and Nef HIV subtype B consensus sequences were computed from all available Gag and Nef sequences from unique patients annotated with Canada (CA) or United States (US) country labels in the Los Alamos HIV sequence database (N = 1624 and N = 1141 Gag and Nef amino acids sequences, respectively, spanning 1983–2011, accessed June 25, 2013). Historic plasma HIV RNA Gag and Nef sequences, annotated with year and country of collection, have been deposited in GenBank (Accession numbers KF701643–KF701941 for Gag and KF701942–KF702276 for Nef).

HLA class I typing was performed using an in-house sequence-based typing protocol capable of using plasma or serum as a source of genomic DNA [48] and types were assigned using an in-house algorithm. Where necessary, data were imputed to high resolution using a machine learning algorithm trained on a dataset of complete high resolution HLA-A, B and C types from >13,000 individuals with known ethnicity ([47]; http://research.microsoft.com/en-us/projects/bio/mbt.aspx#HLA-Completion) and assigned the highest-probability allele combination. HLA types could not be imputed when data were missing from more than one locus.

### Ancestral reconstructions & DNA synthesis

Gag and Nef sequences were annotated with sample dates. Putative recombinants were identified using SCUEAL [102] and removed. The most recent common ancestor (MRCA) sequences of Gag and Nef were estimated using Bayesian evolutionary analysis by sampling trees (BEAST) [52] via 6 (Gag) or 5 (Nef) replicate chains, each analyzing a different set of 200 sequences selected at random from the dataset, and yielding 10,000 ancestral reconstructions per chain, as follows. Trees were sampled at random from the posterior distribution of trees given an exponential relaxed molecular clock [103], a Bayesian skyline model of effective population size, and a time-reversible nucleotide substitution model determined by an Akaike information criterion-based model selection procedure in HyPhy [97]. Sampling was run for 2×10^8^ steps, with the first half discarded as burn-in and the remainder thinned to 100 trees sampled at intervals of 10^6^ steps in the chain. Convergence of replicate chains was assessed using the Tracer application in the BEAST software package. For each tree, 100 ancestral sequence reconstructions were sampled at random from the posterior distribution defined at the root under a Muse-Gaut codon substitution model in HyPhy. The inferred ancestral sequence was taken as the consensus of these 60,000 (Gag) and 50,000 (Nef) reconstructions (10,000 each per chain for 6 [Gag] and 5 [Nef] chains). Timing of each ancestral reconstruction (tMRCA) was estimated in BEAST by computing the mean estimate for each replicate chain and then computing a grand mean. The “consensus ancestor” Gag and Nef nucleotide sequences were commercially synthesized (Invitrogen LifeTech) for use in functional analyses.

### Definition and identification of HLA-associated polymorphisms

The reference list of HLA-associated polymorphisms in modern HIV subtype B sequences was defined in an independent multicenter cohort of >1800 chronically subtype-B infected individuals from Canada, the USA and Australia recruited in the 1990s and 2000s, that did not overlap with historic and modern cohorts analyzed herein, using phylogenetically-informed methods [43]. The same methods [43] were used to identify HLA-associated polymorphisms in the historic dataset, as follows. Briefly, maximum likelihood phylogenetic trees were constructed using Gag and Nef sequences, and a model of conditional adaptation was inferred for each observed amino acid at each codon. Here, the amino acid is assumed to evolve independently along the phylogeny, until it reaches the tree tips (representing the present host). In each host, selection via HLA-mediated pressures and HIV amino acid covariation is directly modeled using a weighted logistic regression, in which the individual's HLA repertoire and covarying amino acids are used as predictors and the bias is determined by the possible transmitted sequences as inferred by the phylogeny [104]. To identify which factors (HLA and/or HIV covariation) contribute to the selection pressure, a forward selection procedure is employed where the most significant association is iteratively added to the model, with p-values computed using the likelihood ratio test. Statistical significance is reported using q-values [105], the p-value analogue of the false discovery rate (FDR). Q-values denote the expected proportion of false positives among results deemed significant at a given p-value threshold; for example, at q≤0.05, we expect 5% of identified associations to be false positives. HLA-associated polymorphisms are grouped into two categories: (1) amino acids significantly enriched in the *presence* of the HLA allele in question (“adapted” forms), and (2) amino acids significantly enriched in the *absence* of the HLA allele in question (“nonadapted” forms).

### Generation of recombinant viruses expressing clonal Gag sequences from patients

Second round Gag amplicons were selected from 120 historic and 71 modern patients with known or presumed chronic infection and cloned into the pCR2.1-TOPO TA vector (Life Technologies, Burlington, ON, Canada). A single representative clone harboring an intact Gag reading frame that closely resembled the patient's bulk plasma HIV RNA was selected for virus generation [60], [61]. Gag was amplified by PCR from each clone using 100 bp-long primers matching the NL4-3 sequence upstream and downstream of Gag, designed to facilitate homologous recombination of the amplicon with the pNL4-3Δ*gag* backbone. The plasmid pNL4-3Δ*gag* was developed by inserting unique BstEII restriction sites at the 5′ and 3′ ends of *gag* using the QuikChange XL kit (Stratagene), followed by deletion of the intervening region via BstEII digestion (New England Biolabs), gel-purification, and re-ligation (T4 DNA ligase; New England Biolabs). PNL4-3Δ*gag* was maintained in Stbl3 *E. coli* cells (Invitrogen). To generate recombinant viruses, 10 µg of BstEII-linearized pNL4-3Δ*gag* plus 50 µl of 2^nd^ round Gag amplicon (∼5 µg) were mixed with 2.5×10^6^ cells of a GFP-reporter T-cell line (CEM-derived GXR25 cells [106]) in 125 µl of Mega-Cell medium (Sigma), and transfected by electroporation in 96-well plates (exponential protocol: 250 Volts, 2000 µF; 25 millisecond pulse duration; BioRad MxCell_Pro). Following transfection, cells were rested for 15 min at room temperature, transferred to 25 cm^2^ flasks containing 1 million GXR cells resuspended in 5 mL of R20+ medium (RPMI 1640 containing 20% FCS, 2 mM L-glutamine, 100 units/mL penicillin, and 100 µg/mL streptomycin), and fed with 5 mL R20+ medium on day 5 and with replacement thereafter. Tat-driven GFP expression, indicating productive HIV infection of GXR cells, was monitored by flow cytometry (Guava 8HT, Millipore) starting on day 12 [60], [61]. Once GFP+ expression exceeded 15% among viable cells, supernatants containing recombinant viruses were harvested and aliquots stored at −80°C. Patient origin of all recombinant viruses was confirmed via sequencing of the Gag region.

### Assessment of Gag recombinant viral replication capacity

Viral titers and replication capacity (RC) assays were performed using GXR25 GFP-reporter T-cells, as described [60], [61]. RC assays were initiated at MOI = 0.003, and included one negative control (uninfected cells only) and one positive control (NL4-3 Gag re-introduced into the NL4-3Δ*gag* backbone using identical methods) per 24-well plate. For each virus, the natural log slope of the percentage (%) of GFP+ cells was calculated during the exponential phase of viral spread (days 3–6). This value was divided by the mean rate of spread of all NL4-3 controls such that RC values <1.0 or >1.0 indicate rates of spread that were slower than or faster than NL4-3, respectively. Each virus was assayed in a minimum of two independent experiments and average RC values are reported.

### Assessment of HLA and CD4 downregulation capacity by clonal Nef sequences

First-round Nef amplicons from 102 historic and 86 modern patients were originally selected and amplified using second round primers featuring EcoRI (forward) and SacII (reverse) restriction sites. Amplicons were PCR-purified (GeneJET PCR Purfication Kit, Thermo Scientific) and cloned into the pIRES2-EGFP expression vector (Clontech) as described in [67], [68]. For each patient, a single representative clone harboring an intact Nef reading frame that closely resembled the original bulk plasma HIV RNA sequence by phylogenetic analysis was selected for functional assessment.

CD4 and HLA class I downregulation activities for each Nef clone were measured using a CEM-SS derived T cell line that expresses high levels of surface CD4 and HLA-A*02 (CEM-A*02), constructed as described in [107]. To assess Nef-mediated CD4 and HLA downregulation, 3×10^5^ CEM-A*02 cells were transfected with 5 µg plasmid DNA encoding Nef protein and GFP by electroporation (BioRad GenePulser MX). Twenty hours later, cells were stained with APC-labeled anti-CD4 and PE-labeled anti-HLA-A*02 antibodies (BD Biosciences) and cell surface expression was measured in transfected (GFP-positive) cells by flow cytometry (Guava easyCyte 8HT, Millipore). For patient-derived Nef clones, the median fluorescence intensity (MFI) of CD4 or HLA-A*02 expression in GFP-positive cells was normalized to the MFI of CD4 or HLA-A*02 expression for the negative control (empty pIRES2-EGFP plasmid) and positive control (*nef* reference sequence SF2, cloned into pIRES2-EGFP) to determine the relative CD4 or HLA-A*02 downregulation capacity. As such, a normalized value of 0.0 indicates no downregulation activity and a value of 1.0 indicates downregulation capacity equivalent to that of the positive control Nef_SF2_. All assays were performed in triplicate and results are presented as the mean of these measurements.

Steady state Nef protein levels were measured by Western blot for the minority of Nef clones that displayed poor (<50%) function for either CD4 or HLA-A*02 downregulation activity, alongside 20 randomly-selected clones with activities above this threshold. A total of 5×10^6^ CEM-A*02 cells were transfected by electroporation with 10 µg of plasmid DNA, and cell pellets were collected 20 hours later for preparation of total cell lysates, using a protocol modified from [107]. Lysates were subjected to SDS-PAGE in duplicate and electro-blotted onto PVDF membrane. To ensure detection of patient-derived Nef, duplicate blots were probed using anti-Nef polyclonal antisera developed from rabbit (NIH AIDS Research and Reference Reagent Program Catalog #2949, [108]) or sheep (ARP 444; NIBSC Center for AIDS Reagents, UK). Actin expression was simultaneously assessed. Band intensities were quantified on an ImageQuant LAS 4000 (GE Healthcare Life Sciences). A total of 15 poorly functional Nef clones whose expression could not be detected by Western Blot were excluded from analysis, as *in vitro* cloning or other defects cannot be ruled out. This left 93 historic and 80 modern Nef clones for analysis.

## Supporting Information

Figure S1 HLA distribution in historic and modern cohorts. HLA-A, B and C alleles with frequencies >0.01 in historic and modern cohorts are shown. Alleles exhibiting significant frequency differences between cohorts are indicated by ** (q<0.05, corresponding to p<0.005) and * (q<0.2, corresponding to p<0.05).
(EPS)

Figure S2 Nef phylogenetic trees incorporating historic, modern and published HIV sequences from North America, colored by era and site. Unrooted Maximum-Likelihood phylogenies constructed from historic and modern Nef sequences are drawn on the same distance scale, colored by era (**Panel A**) and site (**Panel B**). North American HIV sequences retrieved from the Los Alamos (LANL) database are in grey.
(EPS)

Figure S3 Gag and Nef Ancestral and Consensus amino acid sequences. “ANCESTOR_NORTHAMERICA” is the reconstructed ancestral amino acid sequence (same as blue sequence in **Figure 3**). “HISTORIC_COHORT_CONS” is the consensus of our historic cohort sequences. “LANL_CONSB_NORTHAMERICA” is the consensus of all Gag and Nef sequences annotated with “Canada” or “USA” as their country of origin in the Los Alamos HIV Database (see methods). “MODERN_COHORT_CONS” is the consensus of our modern cohort sequences. “LANL_CONSB_GLOBAL” is the most recent (2004) HIV subtype B consensus sequence in the Los Alamos HIV Database. (http://www.hiv.lanl.gov/content/sequence/NEWALIGN/align.html)
(EPS)

Figure S4 Nef residues exhibiting significant diversification over time are biased towards known HLA-associated sites. **Panel A:** Differences in Shannon entropy (Δentropy) between modern and historic sequences are shown for every Nef codon. Red bars indicate significant (p<0.001, q<0.01) entropy differences; blue bars indicate differences that are not significant (p≥0.001). Grey dots designate known HLA-associated sites. Green dots designate sites that display significant evidence of pervasive positive selection (dN/dS>1; posterior probability >0.9). **Panel B:** Same as panel A, but sorted by decreasing magnitude of Δentropy. **Panel C:** Graphical depiction of a 2×2 contingency table stratifying variable (<99% conserved) Nef codons based on their status as HLA-associated (yes vs. no), and whether they exhibited significant Δentropy between modern and historic datasets (p<0.001 [red] vs. not [blue]). Ns are indicated above each bar. **Panel D:** Graphical depiction of a 2×2 contingency table stratifying variable (<99% conserved) Nef codons based on their status as HLA-associated (yes vs. no) and evidence that they are under significant pervasive positive selection (dN/dS>1; posterior probability >0.9 [green] vs. not [black]). Ns are indicated above each bar.
(EPS)

Figure S5 Differences in consensus escape mutant frequencies in persons expressing versus lacking the restricting HLA allele(s), by era. **Panel A:** The frequencies of published HLA-associated polymorphisms, where the polymorphism represents the HIV subtype B consensus residue [43] in historic (1979–1989) and modern (2000+) HIV Gag sequences from individuals expressing the restricting HLA allele(s) are shown as linked pairs. A selection of well-known HLA-associated polymorphisms are labeled with their codons and restricting allele(s). **Panel B:** Consensus frequencies at these sites in historic and modern HIV Gag sequences from individuals *lacking* the restricting HLA allele(s). **Panel C:** Frequencies of published consensus HLA-associated polymorphisms in HIV Nef sequences from historic and modern individuals expressing the restricting HLA allele(s). **Panel D:** Consensus frequencies at these sites in HIV Nef sequences from historic and modern individuals *lacking* the restricting HLA allele(s). All P-values were computed using the Wilcoxon matched-pairs test.
(EPS)

Figure S6 Estimated “percentage of pre-adapted sites” in historic versus modern circulating Gag and Nef sequences. The overall burden of HIV polymorphisms that are “pre-adapted” to an individuals' full HLA class I profile (expressed in terms of % “pre-adapted” sites) for HIV sequences circulating in the historic versus modern eras are shown, for Gag (**Panel A**) and Nef (**Panel B**).
(EPS)

Figure S7 General positive correlation between the frequency of an HLA allele and its associated HIV polymorphisms in the population. Each dot illustrates a single HLA class I allele, colored red, blue and green, for HLA-A, -B, and -C alleles, respectively. As expected, a general positive correlation is observed between the frequency of a given HLA allele in the population (y-axis) and the frequency of its associated HIV polymorphisms in the population. This is true for both the historic (**Panel A**) and modern (**Panel B**) cohorts.
(EPS)

Figure S8 Raw data from Gag and Nef functional assessments. **Panel A:** *In vitro* replication capacities (RC) of recombinant NL4-3 viruses expressing inferred ancestral and global consensus B Gag sequences, along with positive (NL4-3) and negative (cells only) controls. RC of ancestral and consensus are equivalent to that of NL4-3. **Panel B:** Representative flow cytometry plots depicting the ability of the inferred ancestral Nef sequence to downregulate CD4 from the cell surface, along with positive (Nef-SF2) and negative (ΔNef) controls. CD4 downregulation capacity of the inferred ancestral Nef sequences is comparable to that of control strain SF2. **Panel C:** Representative flow cytometry plots depicting the ability of the inferred ancestral Nef sequence to downregulate HLA-A*02 from the cell surface, along with positive (Nef SF2) and negative (ΔNef) controls. HLA-A*02 downregulation capacity of the inferred ancestral Nef sequences is comparable to that of control strain SF2. **Panel D:** Western Blots of SF2 Nef (positive control), ΔNef (negative control), ancestral Nef, global consensus B Nef, and representative patient-derived Nef clonal sequences from modern and historic eras.
(EPS)
